# Supplementary material for: A high-resolution mRNA expression time course of embryonic development in zebrafish
Source: eLife. 2017 Nov 16;6:e30860. doi: 10.7554/eLife.30860 (PMC5690287; doi:10.7554/eLife.30860)
Supplement: Supplementary file 6. [file elife-30860-supp6.zip › biolayout-clusters-files/Cluster212-genes.html]

Cluster212


# Cluster212: Genes

| Ensembl ID | Gene Name | Chr | Start | End | Biotype |
| --- | --- | --- | --- | --- | --- |
| ENSDARG00000056649 | htatsf1 | 10 | 26691146 | 26705530 | protein\_coding |
| ENSDARG00000071060 | naa10 | 23 | 14076270 | 14084356 | protein\_coding |
| ENSDARG00000104837 | nudc | 19 | 14295880 | 14307262 | protein\_coding |
| ENSDARG00000033855 | rqcd1 | 22 | 12726774 | 12738573 | protein\_coding |
| ENSDARG00000068641 | taf10 | 8 | 25792753 | 25797967 | protein\_coding |
| ENSDARG00000016304 | tfdp1b | 1 | 232062 | 246529 | protein\_coding |
| ENSDARG00000053805 | trub1 | 12 | 29921523 | 29929004 | protein\_coding |
